# Dopaminergic control of *ADAMTS2* expression through cAMP/CREB and ERK: molecular effects of antipsychotics

**DOI:** 10.1038/s41398-019-0647-7

**Published:** 2019-11-18

**Authors:** Fulgencio Ruso-Julve, Ana Pombero, Fuencisla Pilar-Cuéllar, Nuria García-Díaz, Raquel Garcia-Lopez, María Juncal-Ruiz, Elena Castro, Álvaro Díaz, Javier Vazquez-Bourgón, Agustín García-Blanco, Emilio Garro-Martinez, Helena Pisonero, Alicia Estirado, Rosa Ayesa-Arriola, Juan López-Giménez, Federico Mayor, Elsa Valdizán, Javier Meana, Javier Gonzalez-Maeso, Salvador Martínez, José Pedro Vaqué, Benedicto Crespo-Facorro

**Affiliations:** 1 Department of Psychiatry, University Hospital Marqués de Valdecilla-IDIVAL, Santander, 39011 Cantabria Spain; 2 Department of Molecular Biology, School of Medicine, University of Cantabria, Santander, 39011 Cantabria Spain; 3 Centro de Investigación Biomédica en Red de Salud Mental (CIBERSAM), Instituto de Salud Carlos III, Madrid, 28029 Spain; 4 Instituto de Neurociencias, UMH-CSIC, Alicante, 3550 Spain; 5 Instituto de Biomedicina y Biotecnología de Cantabria, IBBTEC (Universidad de Cantabria, CSIC, SODERCAN), 39011 Santander, Cantabria Spain; 6 Department of Physiology and Pharmacology, School of Medicine, University of Cantabria, Santander, 39011 Cantabria Spain; 7 Infection, Immunity and Digestive Pathology Group, University Hospital Marqués de Valdecilla-IDIVAL, Santander, 39011 Cantabria Spain; 8 Department of Psychiatry, Sierrallana Hospital, Torrelavega, 39300 Cantabria Spain; 9 Institute of Parasitology and Biomedicine “López-Neyra” (IPBLN-CSIC), Armilla, 18016 Granada Spain; 10 Department of Molecular Biology, Centro de Biología Molecular “Severo Ochoa” (UAM-CSIC), Universidad Autónoma de Madrid, Madrid, 28049 Spain; 11 Centro de Investigación Biomédica en Red de Enfermedades Cardiovasculares (CIBERCV), Instituto de Salud Carlos III, Madrid, 28029 Spain; 12 Department of Pharmacology, University of the Basque Country UPV/EHU, Leioa, 48940 Bizkaia Spain; 13 Department of Physiology and Biophysics, Virginia Commonwealth University School of Medicine, P.O. Box 980551, Molecular Medicine Research Building 5-038, Richmond, 23298 Virginia USA; 14 Department of Psychiatry, School of Medicine, University Hospital Virgen del Rocio-IBiS, Sevilla, 41013 Spain

**Keywords:** Molecular neuroscience, Schizophrenia

## Abstract

A better understanding of the molecular mechanisms that participate in the development and clinical manifestations of schizophrenia can lead to improve our ability to diagnose and treat this disease. Previous data strongly associated the levels of deregulated *ADAMTS2* expression in peripheral blood mononuclear cells (PBMCs) from patients at first episode of psychosis (up) as well as in clinical responders to treatment with antipsychotic drugs (down). In this current work, we performed an independent validation of such data and studied the mechanisms implicated in the control of *ADAMTS2* gene expression. Using a new cohort of drug-naïve schizophrenia patients with clinical follow-up, we confirmed that the expression of *ADAMTS2* was highly upregulated in PBMCs at the onset (drug-naïve patients) and downregulated, in clinical responders, after treatment with antipsychotics. Mechanistically, *ADAMTS2* expression was activated by dopaminergic signalling (D_1_-class receptors) and downstream by cAMP/CREB and mitogen-activated protein kinase (MAPK)/ERK signalling. Incubation with antipsychotic drugs and selective PKA and MEK inhibitors abrogated D_1_-mediated activation of *ADAMTS2* in neuronal-like cells. Thus, D_1_ receptors signalling towards CREB activation might participate in the onset and clinical responses to therapy in schizophrenia patients, by controlling *ADAMTS2* expression and activity. The unbiased investigation of molecular mechanisms triggered by antipsychotic drugs may provide a new landscape of novel targets potentially associated with clinical efficacy.

## Introduction

Antipsychotic drugs (APDs) remain the standard pharmacological treatment for schizophrenia (SCZ) and psychotic disorders, mainly by targeting dopamine neurotransmission (primarily D_2_ receptors)^1–3^. APDs can be classified into typical (i.e., haloperidol) and atypical (i.e., aripiprazole, risperidone or clozapine), the latter prescribed as first-line drugs and/or in refractory patients^4^. Despite the effectiveness of APDs in the clinical realm, there is a marked disparity among patients with respect to symptoms, responses and side effects^5,6^. Whereas 50–60% of patients achieve an optimal degree of clinical improvement of positive symptoms, little to no improvement of negative symptoms or cognitive deficits is common^5^. Overall, estimates suggest that one-fifth to one-half of patients have treatment-resistant SCZ and about 30–60% of these respond to clozapine^6–8^. These evidences suggest that although D_2_ receptors is a direct target for all drugs used to treating SCZ, its blockade may not tackle the primary biological anomaly in a significant percentage of patients^9^. In this respect, the combined D_1_ and D_2_ receptors antagonism has been proposed to have synergic effects, which could account for the atypical clinical effectiveness of clozapine^10^. From a molecular perspective, the effects of APDs may also include the modulation of D_2_ receptor-independent mechanisms through indirect effects, such as, e.g., those related to other metabotropic receptors implicated in the control cAMP-dependent signalling^11^ and/or those related with receptor-biased agonism^4,12^. Downstream of cAMP, the activation of protein kinase A (PKA) promotes phosphorylation of DARPP-32 (a cytosolic protein), which has been associated with the pathophysiology of SCZ^13,14^. In addition, the glutamate hypothesis of SCZ led to explore its potential clinical application as targets for therapy^15^. The investigation of the mechanisms downstream of the receptors targeted by APDs will certainly guide to finding out new explanations for pathophysiological mechanisms and novel approaches for therapy^4^.

A new paradigm has emerged based on the investigation of transcriptional patterns associated with clinical responses in SCZ, raising the likelihood of revealing unknown molecular mechanisms associated with antipsychotic action that may be crucial in accomplishing optimal clinical responses^16,17^. Using an unbiased transcriptome approach, we have previously described a set of genes that are differentially expressed at the onset of SCZ^16^. In addition to this, we also showed significant differential gene expression elicited after a 3-month APD treatment in responder patients^17^. Among the above-mentioned results, *ADAMTS2* was the highest upregulated gene in SCZ patients at the onset of the disease and also was the most significant downregulated gene, back to ‘healthy levels’, in responder patients to treatment with APDs. The observation that the expression of *ADAMTS2* can be modulated by APDs and its association with clinical response, makes it an appealing candidate to investigate its interface with illness pathology and clinical efficacy^17^.

In the present work we initially performed an independent validation using a new cohort of drug-naïve non-affective psychosis patients. We confirmed that *ADAMTS2* expression was significatively overexpressed at diagnosis, and that treatment with APDs reduced their expression levels back to ‘healthy’ values, which, in turn, was associated with clinical responses (positive symptoms). However, the molecular or cellular mechanisms exerted by APDs to regulate *ADAMTS2* expression are unknown. Following a bottom-up strategy starting from the transcriptional data obtained in SCZ cases and taking advantage of neuronal-like cells towards the identification of membrane receptors and the intracellular mechanism controlled by them, we herein provide new evidence that *ADAMTS2* expression is regulated by dopaminergic signalling cascade (D_1_-class receptors) through ERK and cAMP/cAMP response element-binding protein (CREB) activities.

## Materials and methods

### Human samples and study setting

Human samples for this study were obtained from an ongoing epidemiological and 3-year longitudinal intervention programme of first-episode psychosis (PAFIP) conducted at the University Hospital Marques de Valdecilla (Cantabria, Spain) and biological samples were provided by the IDIVAL biobank. The study was approved by the Cantabria Ethics Institutional Review Board, conforming to international standards for research ethics. Patients meeting inclusion criteria and their families provided written informed consent to be included in the PAFIP.

A new independent set of 30 APD-naïve, first-episode non-affective individuals and 10 healthy individuals (without a history of neuropsychiatric disorders) were used to validate gene expression profiles related to clinical response (Table 1).

**Table 1:** Psychopathological characteristics at baseline, at 3 months and clinical changes during the follow-up period. Comparison between aripiprazole and risperidone.

	Total (*N* = 30)		Statistics	*p*	Aripiprazole (*n* = 16)		Risperidone (*n* = 14)		Statistics	*p*
	Mean	SD			Mean	SD	Mean	SD		
**CGI**										
Baseline	6.5	0.6			6.56	0.51	6.43	0.65	*t* = 0.63^c^	0.532
3 Months	1.8	1.2			1.63	1.20	2.00	1.24		
3-Month change from baseline	−4.7	1.2	*z* = 4.83^a^	0.000	−4.90	0.30	−4.47	0.33	*F* = 0.96^d^	0.336
**YMRS**										
Baseline	13.8	6.2			14.25	7.24	13.29	5.01	*t* = 0.42^c^	0.679
3 Months	1.2	2.3			1.56	2.63	0.79	1.93		
3-Month change from baseline	−12.6	6.5	*z* = 4.78^a^	0.000	−12.25	0.59	−13.00	0.63	*F* = 0.75^d^	0.394
**CDSS**										
Baseline	1.5	3.0			2.06	3.82	0.86	1.75	*t* = 1.08^c^	0.288
3 Months	1.2	2.3			1.19	2.34	1.14	2.25		
3-Month change from baseline	−0.3	3.7	*z* = 0.35^a^	0.724	−0.34	0.59	−0.32	0.63	*F* = 0.00^d^	0.985
**BPRS**										
Baseline	70.3	15.0			69.06	16.08	71.85	13.95	*t* = − 0.49^c^	0.627
3 Months	29.9	7.1			29.06	7.07	30.92	7.48		
3-Month change from baseline	−40.4	15.2	*t* = 14.32^b^	0.000	−41.13	1.81	−39.53	2.01	*F* = 0.35^d^	0.561
**SAPS**										
Baseline	15.9	4.2			15.63	3.50	16.07	4.94	*t* = − 0.29^c^	0.775
3 Months	0.6	1.7			0.69	1.89	0.71	1.49		
3-Month change from baseline	−15.4	4.3	*t* = 19.28^b^	0.000	−15.14	0.44	−15.12	0.47	*F* = 0.00^d^	0.975
**SANS**										
Baseline	4.8	6.9			3.47	6.56	5.93	7.43	*t* = − 0.95^c^	0.352
3 Months	3.9	6.6			4.00	6.47	4.93	7.04		
3-Month change from baseline	−0.9	5.6	*z* = 1.14^a^	0.255	−0.07	1.54	−0.36	1.59	*F* = 0.02^d^	0.899

*BPRS* Brief Psychiatric Rating Scale, *CDSS* Calgary Depression Rating Scale for Schizophrenia, *CGI* Clinical Global Impression, *SANS* Scale for the Assessment of Negative Symptoms, *SAPS* Scale for the Assessment of Positive Symptoms, *YMRS* Young Mania Rating Scale

^a^Wilcoxon matched-pairs signed-rank test

^b^Paired Student’s t-test

^c^Comparison between aripiprazole and risperidone at baseline; unpaired Student’s *t*-test

^d^Comparison between aripiprazole and risperidone following the antipsychotic treatment, using the total score of the clinical scales at baseline as covariate; analysis of covariance (ANCOVA)

Briefly, all patients underwent a head-to-head risperidone and aripiprazole randomized (simple randomization procedure), flexible-dose, open-label study (EudraCT number 2013-005399-16). More detailed information about PAFIP and treatment protocol has been published elsewhere^17,18^ and also is briefly available in Supplementary Information.

### RNA extraction

Peripheral blood from patients and controls was extracted using the TempusTM Blood RNA Tube (Applied Biosystems, UK). Peripheral blood mononuclear cell (PBMC) isolation was performed using a Ficoll-Paque Premium reagent (Sigma) and total mRNA isolation using a TempusTM Spin RNA Isolation Kit (Invitrogen, CA, USA), following the manufacturer’s instructions. RNA Integrity Number (RIN) was characterized with a Bioanalyzer (Agilent Technologies) and samples with a RIN > 8 were selected. Total mRNA from culture cells was extracted using Trizol reagent (Invitrogen) and its concentration was determined using a Nanodrop 2000 spectrophotometer (Thermo Scientific, IL, USA).

### RT-PCR and quantitative PCR

cDNA synthesis was performed using the SuperScript IV Reverse Transcriptase (Invitrogen). cDNA was amplified using the Power SYBR™ Green PCR Master Mix (Applied Biosystems) in a 7300 Fast Real-Time PCR System (Applied Biosystems). Specific oligos for human *ADAMTS2*, *CREB1*, and *C-FOS* were designed using Primer-Blast (NCBI; see sequences in Supplementary Table S1). *ACTB* expression was used to normalize values. Gene expression changes were determined using 2^(–ΔΔCt)^ formula. A melting curve was generated for every run to confirm assay specificity.

### Cell culture and treatments

Human neuroblastoma (SK-N-SH, ATCC HTB-11) and 293T (ATCC CRL-3216) cells were obtained from the American Type Cell Collection (Rockville, MD). Cells were cultured in modified Eagle’s medium and Dulbecco’s Modified Eagle’s Medium, respectively (Corning, VA, USA). Culture medium was supplemented with 10% dialysed fetal bovine serum (dFBS; HyClone, UT, USA), glucose, l-glutamine, streptomycin sulphate and potassium penicillin (10,000 U/L) (Lonza, Belgium).

### Drugs and pharmacological agents

Aripiprazole, clozapine, haloperidol hydrochloride, H89 dihydrochloride, L 741,626, MDL 100907, paliperidone, risperidone, SKF 83822, SCH 39166, TCB-2, WAY 100635, 7-OH-DPAT and 8-OH-DPAT were purchased from Tocris Bioscience (Spain). Forskolin and selumetinib (AZD6244) were purchased from Selleckchem (Spain). Cholera toxin (CTX), pertussis toxin (PTX) and 12-O-tetradecanoylphorbol-13-acetate (TPA) were purchased from Sigma-Aldrich (MO, USA). YM-254890 was purchased from Adipogen Life Sciences (CA, USA). All were dissolved in dimethyl sulfoxide, except CTX and WAY 100635 that were dissolved in water.

### Luciferase report assays

Luciferase report assays were performed by transfection with 0.5 µg DNA of the following plasmids mix (ratio 3:1): pGL4.29[luc2P/CRE/Hygro] containing firefly luciferase reporter, alongside pRL-Null containing *Renilla* luciferase used as control (Promega, WI, USA). Cells were transfected with Lipofectamine LTX with PLUS reagents (Invitrogen) in transient conditions. Firefly and *Renilla* levels were detected using Dual-Luciferase Reporter Assay System kit and quantified using a GloMax-Multi apparatus (Promega).

### Western blotting

Cells were starved overnight before treatment. Whole cell lysates were obtained using RIPA buffer (Sigma) supplemented with phosphatase and protease inhibitors (Roche, Germany). Protein expression was analysed by western blotting as described previously^19^. Briefly, antibodies used were as follows: phospho-CREB, CREB, phospho-ERK1/2, ERK1/2, phospho-p38, p38, and phospho-PKA substrates (Cell Signaling, MA, USA), and β-Tubulin (Santa Cruz Biotechnology). Fluorophore conjugate antibodies were obtained from Invitrogen. Signals were visualized and recorded with an Odyssey Infrared Imaging scanner (LI-COR Biosciences, NE, USA). Immunoblot densitometry analysis on every band was calculated using Image Studio Software (LI-COR Biosciences). Phosphorylation and total protein densitometry values were normalized to β-Tubulin signal.

### CREB knocked down reagents and procedures

ShCREB1-inducible SK-N-SH cells were generated by lentiviral infection of SK-N-SH cells with SMARTvector carrying tGFP and human inducible *CREB1* short hairpin RNA (shRNA) mCMV constructs or non-target control shRNA (Dharmacon, CO, USA). Lentiviral particles were produced by co-transfection of 293T cells using Trans-Lentiviral shRNA Packaging System (Dharmacon), according to the manufacturer’s protocol. Cells were incubated with doxycycline (1 μg/ml) (Sigma) for 72 h to induce green fluorescent protein and shRNA expression. SK-N-SH cells expressing stable shCREB1 constructs were generated by direct transfection by Lipofectamine LTX with PLUS reagent (Invitrogen) using pGFP-V-RS-CREB1 shRNA expression vectors or scrambled control (Origene, MD, USA). Transfected cells were selected with puromycin (1 μg/mL) at least 7 days.

### Statistical analyses

Kolmogorov–Smirnov test and Levene test were used to test normality and equality of variances, respectively.

For patients’ studies, to ensure group comparability between healthy volunteers and patients, sociodemographic and clinical characteristics at baseline were tested by unpaired Student’s *t*-test or one-way analysis of variance (ANOVA) for continuous variables as necessary, and by Fisher’s exact test for qualitative variables (Table 1 and Supplementary Table S2).

Wilcoxon’s signed-rank test for independent data was used to compare the *ADAMTS2* mRNA expression level among healthy volunteers and drug-naïve patients at baseline. Wilcoxon’s matched-pairs signed-rank test for paired data was performed to compare the change in the mRNA expression level from baseline to 3 months following the antipsychotic treatment (*α* = 0.05). STATA 15.1 was used for statistical analysis.

For in vitro studies, unless otherwise specified, all experiments were independent and numerical data were summarized as the mean ± SEM using GraphPad Prism6 software. Each global mean was compared using unpaired Student’s *t*-test (two tailed; *α* = 0.05) or one-way ANOVA followed by post-hoc test where appropriated, as described in each figure legend.

## Results

### *ADAMTS2* expression is controlled by APD treatment in SCZ patients

To better evaluate the mechanistic effects of atypical APDs in SCZ patients, we prepared and studied a new cohort of 30 drug-naïve SCZ patients with baseline quantitative data (sociodemographic and clinical characteristics) and clinical follow-up data, after 3 months of treatment with APDs (Table 1). We found significant within-subject changes between baseline data and after 3 months of treatment in both risperidone and aripiprazole groups. These changes were observed in CGI (Clinical Global Impression, *p* < 0.000), YMRS (Young Mania Rating Scale, *p* < 0.000), BPRS (Brief Psychiatric Rating Scale, *p* < 0.000) and SAPS (Scale for the Assessment of Positive Symptoms, *p* < 0.000) total scores. However, as it is shown in Table 1, no differences were found in total scores of the clinical scales when the two APD groups were compared at baseline and after 3 months of treatment.

**Fig. 1:**
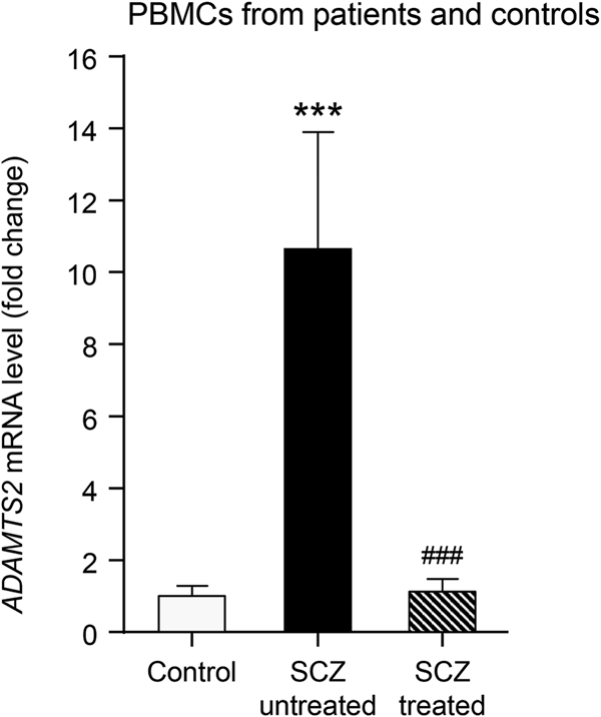
Transcriptional control of *ADAMTS2* in SCZ patients at onset and after 3-month antipsychotic treatment. Relative mRNA expression level of *ADAMTS2* gene in PBMCs from peripheral blood samples of an independent cohort of SCZ patients: healthy controls (basal; grey bar), untreated FEP patients (basal drug-naïve; black bar) and 3-month treated patients (striped bar). FEP: first episode of psychosis. All patients were treated with risperidone or aripiprazole drugs and showed clinical response to treatment (Cohort: patients *N* = 30 and controls *N* = 10). Data are mean ± SEM; unpaired Wilcoxon’s signed-rank test: ***p < 0.001 compares patients at baseline *vs.* healthy controls; paired Wilcoxon’s signed-rank test: ###*p* < 0.001 compares patients at baseline *vs.* those after 3-month AP treatment.

To analyse transcriptomic changes, we obtained PBMCs isolated from blood samples from SCZ patients, which were obtained at the first episode of psychosis (onset) and after a 3-month period of treatment with APDs. In this context, using quantitative reverse transcriptase PCR (RT-qPCR), we performed an independent validation of transcriptional changes by focusing on *ADAMTS2*. Thus, we confirmed *ADAMTS2* as a significant gene that was highly overexpressed at the onset, with respect to healthy controls, and downregulated by APDs after 3 months of treatment (Fig. 1).

### Dynamic control of *ADAMTS2* transcription by APDs in neuronal-like cells

**Fig. 2:**
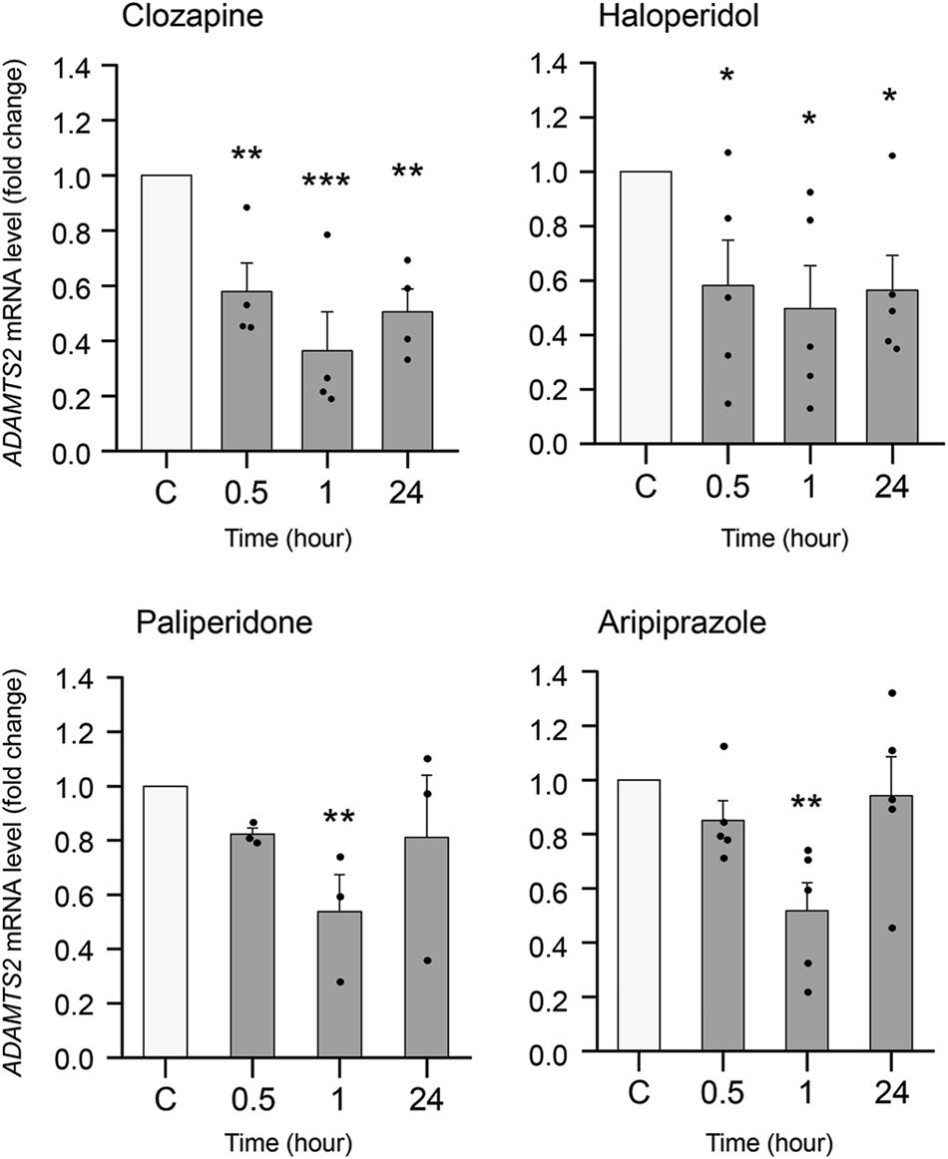
Transcriptional control of *ADAMTS2* using antipsychotic drugs. Relative *ADAMTS2* mRNA expression level in SK-N-SH cells incubated with clozapine (1 µM) (*N* = 4), haloperidol (1 µM) (*N* = 5), paliperidone (1 µM) (*N* = 3) and aripiprazole (1 µM) (*N* = 5) for the indicated times. Data are mean ± SEM; one-way ANOVA for multiple comparations: *p < 0.05, **p < 0.01, ***p < 0.001 shows significance with respect to control (C; vehicle, grey bars).

To gain mechanistic insight into the transcriptional control of *ADAMTS2*, we analysed the effects of APDs in SK-N-SH cells using dFBS. No significant differences in *ADAMTS2* expression were observed when dFBS was not present in the culture medium (data not shown). These cells express cell surface receptors that are targets of APDs (i.e., D_1_, D_2_, 5-HT_1A_ and 5-HT_2A_ receptors) as well as detectable basal expression of *ADAMTS2* mRNA. Our data showed that incubation with APDs in these cells induced dynamic changes in *ADAMTS2* mRNA expression along time. Incubation with atypical APDs such as paliperidone, aripiprazole (Fig. 2) and risperidone (Supplementary Fig. S1), provoked a dynamic inhibition of *ADAMTS2* basal mRNA expression in these cells with fast (<1 h) but transitory responses. Interestingly, clozapine (atypical APD) and haloperidol (typical APD) induced a fast and sustained (up to 24 h) inhibition of *ADAMTS2* expression (Fig. 2).

### Neurotransmitter receptors and associated signalling pathways involved in the control of *ADAMTS2* gene expression

**Fig. 3:**
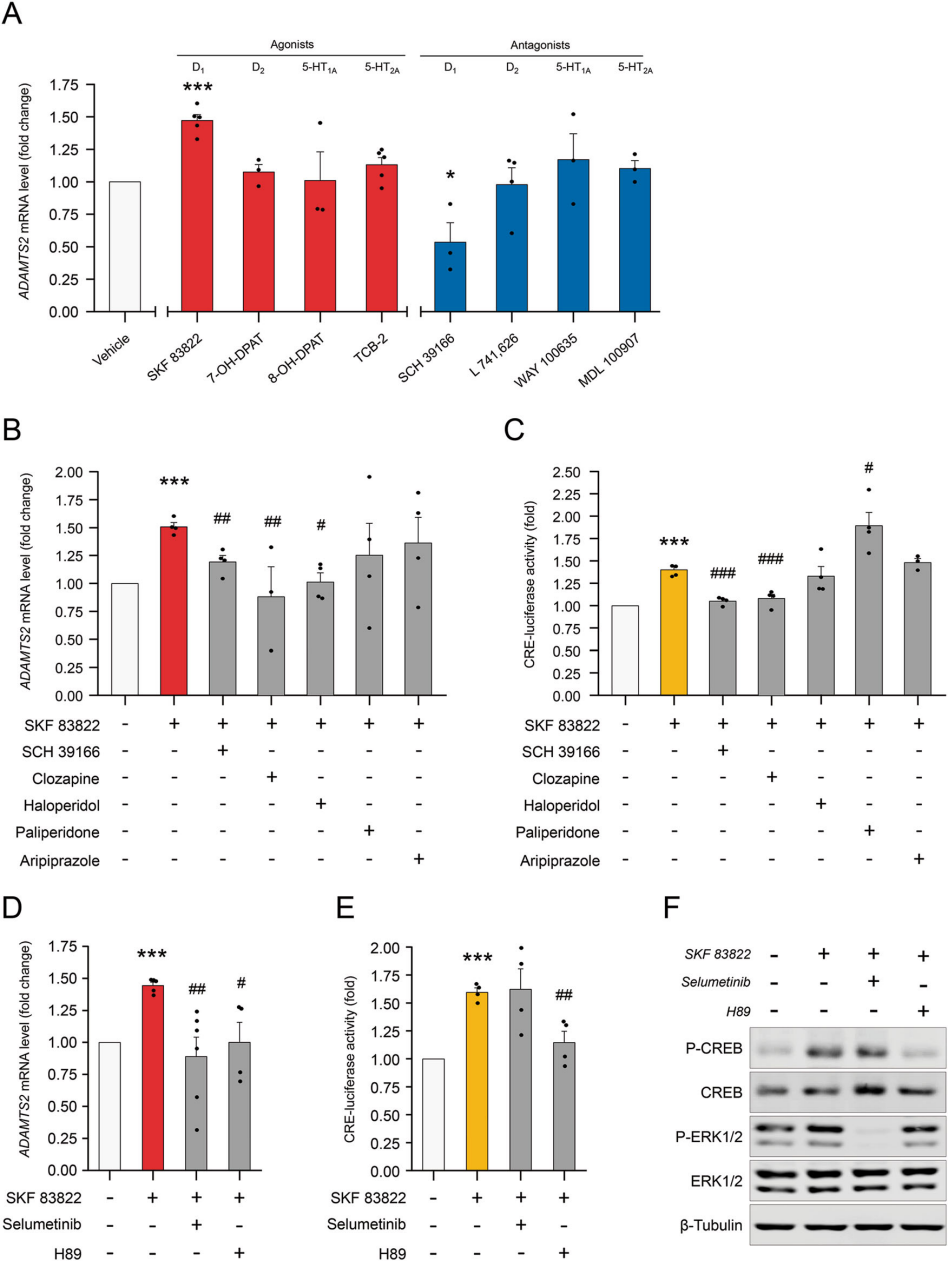
Neurotransmitter receptors and associated signalling pathways involved in the control of *ADAMTS2* gene expression. **a** *ADAMTS2* mRNA levels by RT-qPCR in SK-N-SH cells incubated 1 h with the indicated selective receptor agonist (red bars): SKF 83822 (D_1_-class receptors) (*N* = 5), 7-OH-DPAT (D_2_-class receptors) (*N* = 3), 8-OH-DPAT (5-HT_1A_ receptor) (*N* = 3) and TCB-2 (5-HT_2A/2C_ receptors) (*N* = 5); and selective antagonist (blue bars): SCH 39165 (D_1_-class receptors) (*N* = 3), L 741,626 (D_2_-class receptors) (*N* = 4), WAY 100635 (5-HT_1A_ receptors) (*N* = 3) and MDL 100907 (5-HT_2A_ receptors) (*N* = 3) (Drug concentration 1 µM). **b** *ADAMTS2* mRNA levels by RT-qPCR in cells incubated for 1 h with SKF 83822 (*N* = 4) and pre-incubated also for 30 min with SCH 39166 (*N* = 4), clozapine (*N* = 3), haloperidol (*N* = 4), paliperidone (*N* = 4) or aripiprazole (*N* = 4) (Drug concentration 1 µM). **c** CREB activity in cells transfected with CRE-Luc alongside pRL-Null: cells were pre-incubated for 1 h with the indicated APDs and then, incubated for 24 h with SKF 82833 (10 µM) (*N* = 4). **d** *ADAMTS2* mRNA levels by RT-qPCR: SK-N-SH cells were pre-incubated for 30 min with MAPK/ERK and cAMP-PKA inhibitors (selumetinib 1 µM *N* = 6 and H89 10 µM N = 4, respectively) and then, incubated for 1 h with SKF 82833 (1 µM) (*N* = 5). **e** CREB activity in cells transfected with CRE-Luc alongside pRL-Null (*N* = 4): SK-N-SH cells were pre-incubated for 1 h with the indicated inhibitors and then incubated for 24 h with SKF 82833 (10 µM). **f** Western blottings showing relative phosphorylation levels of CREB and ERK1/2: SK-N-SH cells were pre-incubated for 1 h with the indicated inhibitors and then incubated for 15 min with SKF 82833 (1 µM) (*N* = 3). Blots are representative images of each western-blot. Data are mean ± SEM; Student’s *t*-test: *p < 0.05 and ***p < 0.001 *vs.* control condition (vehicle), and #*p* < 0.05, ##*p* < 0.01, ###*p* < 0.001 *vs.* SKF 83822 condition.

Using selective agonists for D_1_, D_2_, 5-HT_1A_ and 5-HT_2A_ receptors in SK-N-SH cells, we observed that SKF 83822 (a D_1_-class receptor agonist) significantly triggered *ADAMTS2* mRNA expression compared with the lack of effect of 7-OH-DPAT, 8-OH-DPAT and TCB-2 (agonists of D_2_, 5-HT_1A_ and 5-HT_2A_ receptors, respectively) (Fig. 3a). In addition, using selective antagonists for these receptors, we observed that only SCH 39166 (a D_1_-class receptor antagonist) significantly downregulated *ADAMTS2* mRNA expression, whereas L 741,626, WAY 100635 and MDL 100907 (antagonists of D_2_, 5-HT_1A_ and 5-HT_2A_ receptors, respectively) were devoid of this effect (Fig. 3a). Furthermore, we analysed *ADAMTS2* mRNA and protein expression profiles in the brain of developing and early postnatal mice (see Supplementary Methods). Interestingly and in support of our previous results in cells, ADAMTS2 was specifically localized in brain regions that are part of the mesolimbic and mesocortical dopamine systems (Supplementary Fig. S2).

Next, we studied whether treatment with APDs could modulate *ADAMTS2* gene expression activated downstream of the D_1_ receptor. Our results show that haloperidol and clozapine could significantly block SKF 83822-mediated transcriptional activation of *ADAMTS2*. As expected, SCH 39166, a selective D_1_ receptor antagonist, also prevented D_1_ receptor-mediated activation of *ADAMTS2* (Fig. 3b). In addition, the reduction in *ADAMTS2* expression induced by clozapine was prevented by L 741,626 and MDL 100907 (a D_2_ and 5-HT_2A_ receptor antagonists, respectively) (Supplementary Fig. S3).

As D_1_ receptors are known to signal through G_αs_, we sought to confirm its role at controlling *ADAMTS2* expression in our system, alongside G_αi_ and G_αq_. For this purpose, we incubated SK-N-SH cells with CTX (G_αs_ activator), PTX (G_αi_ inhibitor) and YM-254890 (a specific G_αq_ inhibitor)^20^, before activating D_1_ receptors with SKF 83822. The activation of G_αs_ (Supplementary Fig. S4A) and the inhibition of G_αi_ (Supplementary Fig. S4B) per se significantly upregulated *ADAMTS2* expression. Moreover, the inhibition of G_αi_ potentiated *ADAMTS2* expression after D_1_ receptor activation (Supplementary Fig. S4B). Finally, the inhibition of G_αq_ did not modify the basal and the SKF 83822-mediated increase of *ADAMTS2* expression (Supplementary Fig. S4C).

Incubation of neuronal-like cells with the selective D_1_ receptor agonist (SKF 83822) provoked rapid phosphorylation of CREB and ERK1/2 (15 min), and no changes in p38 (Supplementary Fig. S4D-F). In addition, rapid phosphorylation of PKA substrates was detected in response to SKF 83822 (Supplementary Fig. S4G). To evaluate CREB-dependent transcriptional activity in neuronal-like cells, we took advantage of a CRE-luciferase reporter. We analysed if APDs could counteract the intracellular signalling activation mediated by selective SKF 83822 D_1_-class receptor agonist. Our results (Fig. 3c) demonstrate that preincubation with clozapine, but not haloperidol, paliperidone, or aripiprazole, prevented SKF 83822-induced CREB activation using a specific reporter assay.

To evaluate the potential contribution of PKA and ERK to the activation of *ADAMTS2* expression, we incubated SK-N-SH cells with SKF 83822 D_1_ receptor agonist in combination with selective MAPK/ERK (selumetinib-AZD6244) and cAMP/PKA (H89 dihydrochloride) inhibitors. Both inhibitors prevented D_1_ receptor induced expression of *ADAMTS2* (Fig. 3d), although only a cAMP/PKA inhibitor abrogated CREB-dependent transcription and phosphorylation (Fig. 3e, f).

### Transcriptional mechanisms controlling *ADAMTS2* gene expression downstream of dopamine D_1_ receptors

**Fig. 4:**
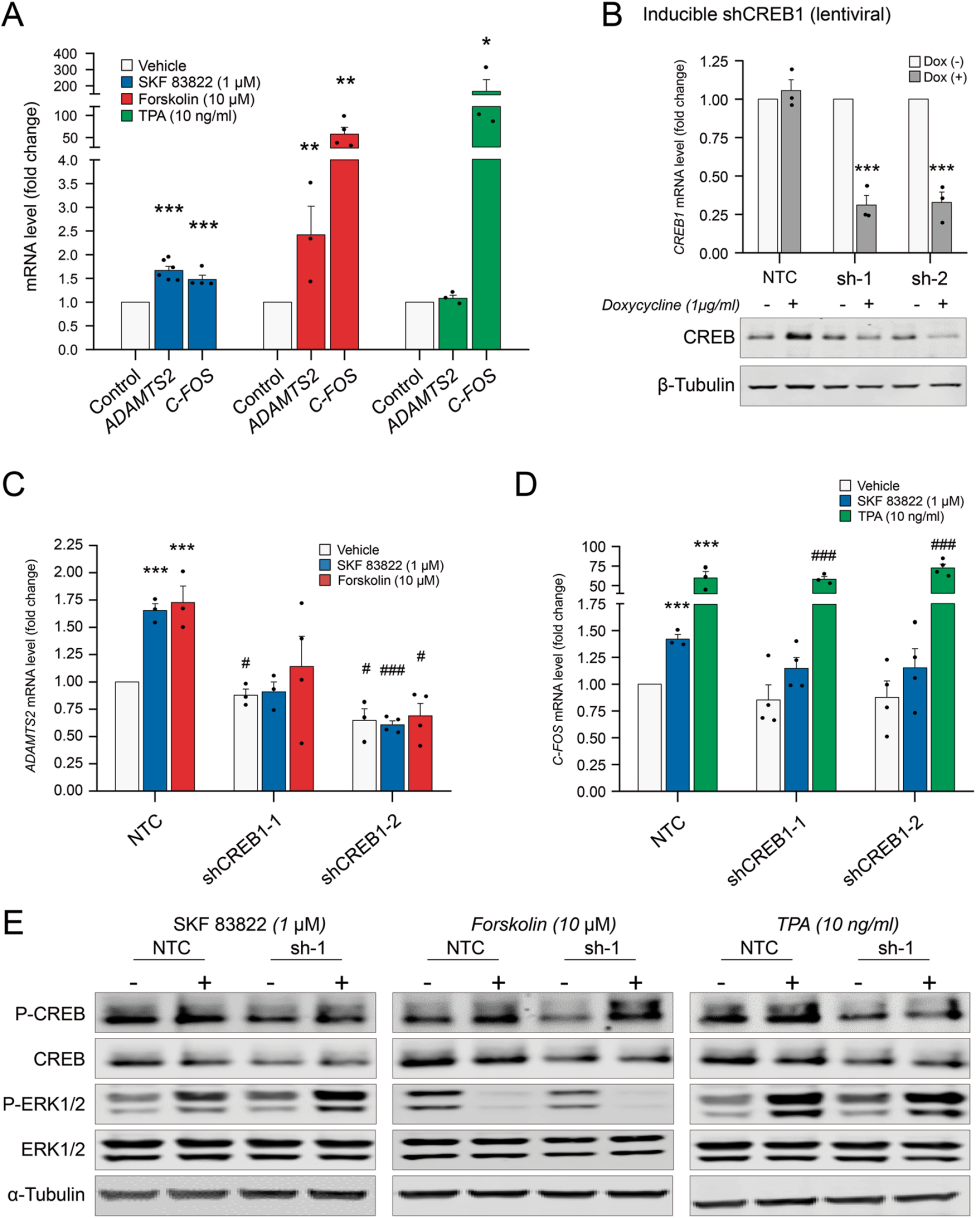
Transcriptional mechanisms that control *ADAMTS2* gene expression downstream of dopamine D_1_-class receptors. **a** RT-qPCR showing *ADAMTS2* and *C-FOS* mRNA expression level in SK-N-SH cells incubated for 1 h with SKF 83822 (1 µM; blue bars) (*N* = 6 and *N* = 4, respectively), forskolin (10 µM; red bars) (*N* = 3 and *N* = 4, respectively) and TPA (10 ng/ml; green bars) (*N* = 3). **b** *CREB1* knockdown SK-N-SH cells by lentiviral inducible shRNA and GFP reporter construct (yellow bars), incubated with doxycycline (1 µg/ml) for 72 h, *CREB1* mRNA level (up) and CREB protein total expression (down) in non-targeted control (NTC) or shCREB1 cells (*N* = 3). Inducible *CREB1* Knockdown SK-N-SH cells were incubated for 1 h with SKF 83822 (1 µM, blue bars), forskolin (10 µM, red bars) and TPA (10 ng/ml, green bars): RT-qPCR showing *ADAMTS2* (**c**) and *C-FOS* (**d**) mRNA expression in inducible shCREB1 or non-targeted control (NTC) cells incubated with doxycycline (1 µg/ml) for 72 h (*N* = 3-4). **e** Shows western blottings using anti-phospho-CREB and anti-phospho-ERK, as well as anti-CREB and anti-ERK antibodies: inducible shCREB1 or non-targeted control (NTC) SK-N-SH cells incubated with doxycycline (1 µg/ml) for 72 h and then incubated for 15 min with SKF 83822 (1 µM), forskolin (10 µM) and TPA (10 ng/ml) (*N* = 3). Inducible knockdown cells were selected with puromycin (1 µg/ml) at least 7 days. Blots images are representative of independent experiments. Data are mean ± SEM; Student’s *t*-test: *p < 0.05, **p < 0.01, ***p < 0.001 *vs*. vehicle or doxycycline (−); and #*p* < 0.05, ###*p* < 0.001 *vs*. each condition in NTC cells.

Our previous data suggest that there is a rapid activation of the signalling mechanisms controlling CREB activation after D_1_ receptor activation that can be modulated pharmacologically. We decided to challenge the contribution of cAMP/CREB signalling to the transcriptional activation of *ADAMTS2* (Fig. 4a). We initially evaluated the activation of *ADAMTS2* gene expression together with that of *C-FOS*, an ‘early response gene’ known to be a direct transcriptional target of CREB that was used as control. Our data showed that SKF 83822 activated mRNA expression of both *ADAMTS2* and *C-FOS*. Interestingly, incubation with forskolin alone (a selective adenyl cyclase activator) was sufficient to trigger the transcription of both genes, whereas TPA (a PKC/MAPK activator) uniquely activated *C-FOS* transcription.

**Fig. 5:**
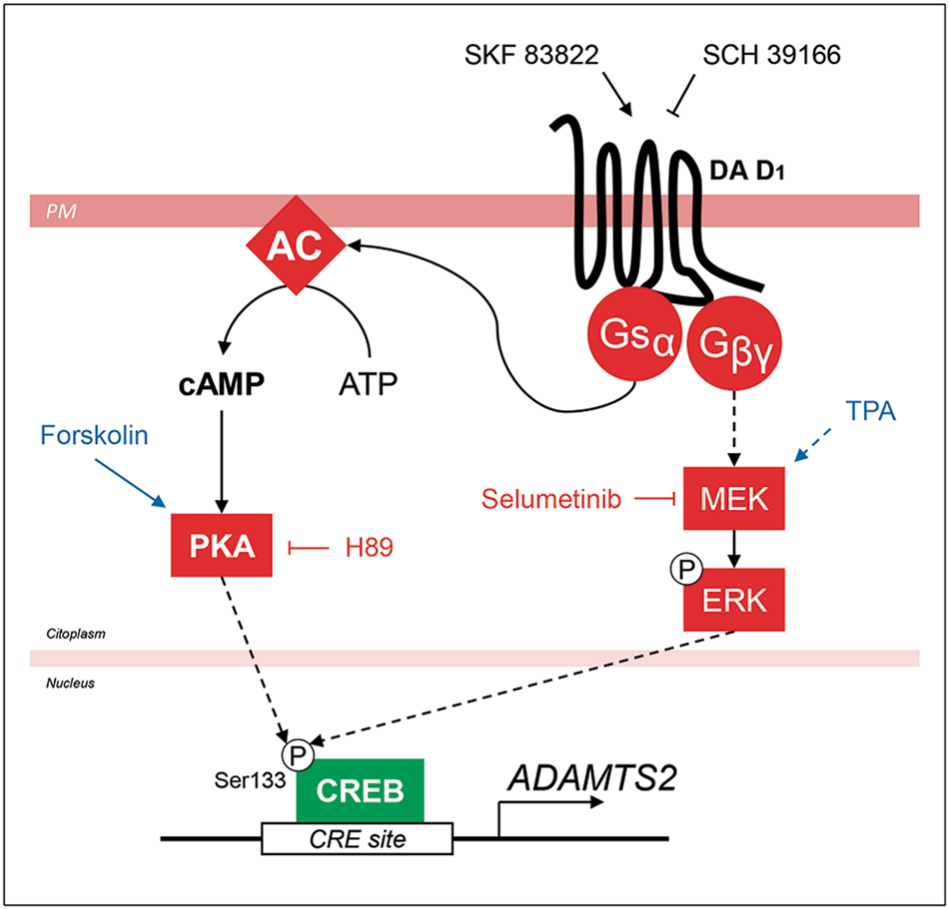
Schematic representation of the mechanisms that control *ADAMTS2* gene expression. Selective stimulation of D_1_ receptors by SKF 83822 (selective D_1_ receptor agonist) triggers the expression of *ADAMTS2*. Two main pathways seem to be involved: (1) G_αs_/AC/cAMP/PKA signalling and (2) MEK/ERK1/2 signalling. Downstream of D_1_ both PKA and ERK can phosphorylate CREB at Ser133 and activate transcription of *ADAMTS2*. Specific activators of PKA (Forskolin) and MEK (TPA) are highlighted in blue. Specific inhibitors of PKA (H89) and MEK (selumetinib) are coloured in red. DA D_1_ (dopamine D_1_ receptor), Gsα (G-protein α-subunit), Gβγ (G-protein βγ-subunits), AC (adenyl cyclase), PKA (protein kinase A), CRE (cyclic AMP-responsive element) site and PM (plasmatic membrane). Arrows: direct interaction, dashed arrows: indirect interaction.

To explore the implication of CREB in D_1_ receptor-mediated *ADAMTS2* expression, we generated stable SK-N-SH cells with inducible expression of control or *CREB1* shRNAs upon addition of doxycycline to the culture media. Using this approach, we reduced CREB1 mRNA and protein expression, as shown in Fig. 4b. Under these settings, SKF 83822 and forskolin failed to induce the expression of *ADAMTS2* (Fig. 4c). Interestingly, SKF 83822 did not significantly activate *C-FOS* expression in *CREB1* knocked down cells when compared with control cells; in contrast, TPA did (Fig. 4d). A schematic representation of the signalling pathways and the molecules used in this work is illustrated in Fig. 5 for explanatory purposes. The above-mentioned results were further confirmed using SK-N-SH cells stably expressing control or CREB1 shRNAs (Supplementary Fig. S5). Mechanistically, reduced CREB expression did not impair ERK phosphorylation elicited downstream of D_1_ receptor activation by SKF 83822 (Fig. 4e). In this context, we also used forskolin and TPA as controls for selective activation of CREB and ERK, respectively (Fig. 4e and Supplementary Fig. S6). Thus, activation of CREB seems to play a key role in the control of *ADAMTS2* gene expression downstream of D_1_ receptors.

## Discussion

Our results revealed that treatment with APDs control *ADAMTS2* expression, which is directly associated with dopaminergic signalling, primarily with the D_1_-class receptors and downstream through cAMP/CREB and MAPK signalling. Interestingly, *ADAMTS2* mRNA and protein were specifically found in mesolimbic and mesocortical dopaminergic regions in mice. Our data suggest that D_1_ receptor signalling towards CREB activation and its effects on *ADAMTS2* expression may be linked to key biological mechanisms in SCZ and the clinical response to APDs^21,22^.

In the context of the local PAFIP programme, we collected a new cohort of 30 drug-naïve SCZ patients with associated clinical data and follow-up. Using these cases alongside ‘healthy’ controls, we attempted an independent validation of our previous results using a different cohort of cases^16,17^. In this cohort, we confirmed *ADAMTS2* as a significant gene that was highly overexpressed at baseline in PBMCs from drug-naïve SCZ individuals and which returned to ‘normal’ levels in clinical responders treated with APDs (clinical rate scales in Table 1). Although, there is not always a direct correlation between the biomarkers obtained in peripheral blood cells and the central nervous system (CNS) samples, a previous study in a rodent model of SCZ have shown a certain parallelism between the expression in human PBMCs^16,17^ and mouse frontal cortex samples^23^. Supporting the previously mentioned data, *ADAMTS2* mRNA expression was quickly downregulated by all APDs in neuronal-like cells. Moreover, whereas the activities of paliperidone, risperidone, and aripiprazole downregulating *ADAMTS2* mRNA were transitory in such cells, those of clozapine and haloperidol were sustained to up to 24 h. The molecular effects exerted by clozapine and haloperidol as compared with other APDs might deserve further investigations. In this respect, clozapine has shown superior efficacy for treatment-resistant and suicidality, as well as its apparent ability to decrease substance use in SCZ^24^. Thus, ADAMTS2 might play a key, and yet to be defined, mechanistic role in both the illness onset and clinical responses, regardless of the type of APD used.

There is scarce information regarding specific *ADAMTS2* expression and activity in the CNS. Therefore, we analysed the expression profiles of *ADAMTS2* mRNA and protein in mice. Interestingly, these were specifically localized in brain regions that are part of the mesolimbic and mesocortical dopamine systems (i.e., the dentate gyrus in the hippocampus and the ventral tegmental area^21,22,25^. At a prenatal stage (E18.5), ADAMTS2 protein was mapped in the neuropiles of anterior brain structures, anterior cingulate cortex, superficial striatum and lateral septum, where neuronal expression of dopamine D_1_ and D_2_ receptors was detected by in situ hybridization (http://developingmouse.brain-map.org/). The hypothesis that dopamine and dopaminergic mechanisms are essential to psychosis, and particularly to SCZ, has been one of the most enduring ideas about this disorder^26^. Elevated presynaptic striatal dopamine correlates most closely with the symptom dimension of psychosis and blockade of this heightened transmission leads to a resolution of symptoms for most patients^27^. Nonetheless, the functional association between *ADAMTS2* and the dopaminergic system has not been previously established, to our best knowledge. ADAMTS2 is a member of the ADAM Metallopeptidase with Thrombospondin family^28,29^, with a number of targets such as the *N*-propeptides of procollagens I–III, fibronectin, decorin and Dkk3 participating in extracellular matrix (ECM) organization, as well as in transforming growth factor (TGF)-β and WNT signalling^30,31^. Alterations in the ECM as well as TGF-β and WNT signalling have been associated with SCZ^32–34^. Moreover, deregulated mRNA expression of MMPs (MMP-16, -24 and -25) and ADAMTS (ADAMTS-1, -6 and -8) families of proteases have also been reported in SCZ^35,36^. Thus, considering *ADAMTS2* expression and its associated activities in the CNS, as well as the data presented in this work, it is conceivable to speculate that it could participate in SCZ at different stages of the disease. In this regard, a selective D_1_-class receptor agonist (SKF 83822) significantly activated *ADAMTS2* expression, and haloperidol and clozapine blocked this activation in neuronal-derived cells. It will be of interest, to address these appealing questions, within the next future, using SCZ and/or *ADAMTS2* transgenic mice models.

D_1_ receptors are the most abundant dopaminergic receptor in CNS and their functional crosstalk with D_2_ receptor is well documented^11,37,38^. D_1_ receptor activate adenylyl cyclase (AC), which in turn regulate intracellular cAMP levels leading to PKA activation and CREB phosphorylation^39–41^. Our data have shown that selective D_1_ receptor activation upregulated *ADAMTS2* expression alongside a rapid phosphorylation of CREB and ERK proteins, which resulted in CREB-mediated transcriptional activity as detected by using specific reporter assays (Fig. 3). In support of this and in our system, *ADAMTS2* transcription was specifically triggered by activation of D_1_ receptors (by SKF 83822), G_αs_ (using CTX) and AC (forskolin). Thus, it is possible that, as part of the dopaminergic activity, *ADAMTS2* could act as a major cAMP/CREB effector in SCZ. Interestingly, D_1_ receptor-mediated CREB activation was abrogated by clozapine (Fig. 3c), providing evidence that cAMP/CREB signalling, and therefore *ADAMTS2* expression, can be modulated by APDs. Our findings reveal the contribution of D_1_ receptors over *ADAMTS2* transcription, and also indicate the participation of D_2_ and 5-HT_2A_ receptors in the effect of clozapine in the control of *ADAMTS2* expression. In this regard, it has been reported that clozapine can act as a biased agonist on 5-HT_2A_ receptors^42,43^ and/or affect the hetero-dimer D_2_/5-HT_2A_^44^. Moreover, disrupting cAMP/CREB and ERK signalling using selective PKA and MEK inhibitors also impaired *ADAMTS2* expression and CREB activity (in this case, only when it was PKA dependent) in neuronal-like cells. Supporting these observations, recent data pointed at cAMP/CREB signalling as an important mechanism linking dopaminergic signalling with the pathophysiology of SCZ^45^. Moreover, incubating SK-N-SH cells with forskolin was sufficient to trigger *ADAMTS2* expression that occurred alongside PKA and CREB activation. Forskolin directly activates AC in mammals, thereby promoting a rapid phosphorylation of CREB via PKA^46,47^. In addition, in CREB-knockdown cells, D_1_ receptor activation failed to increase *ADAMTS2* gene expression, reinforcing the idea that cAMP/CREB is an essential mechanism to control *ADAMTS2* transcriptional activation.

Finally, a number of potential limitations could be considered when interpreting our data and the scope of our findings: (1) sample size in this study is rather small (*N* = 30). Noteworthy, we selected a group of patients with a first episode of non-affective psychosis, who had not previously taken APDs (not even a single dose) at the time of baseline blood test to avoid any interference with the mRNA levels. Only those individuals who gave written consent and had mRNA samples at baseline and at 3 months were eligible for this study. Therefore, all these stringent inclusion criteria limited the number of patients in this cohort. Further investigations to replicate our findings using larger and more heterogeneous groups are warranted. (2) Transcriptomic data obtained from blood samples might not resemble that of the brain. (3) *ADAMTS2* protein and mRNA expression show modest but detectable levels in ‘healthy’ human and mouse brain samples (https://www.proteinatlas.org). Thus, it is conceivable to detect high fold increases when analysing diseased specimens, like in this case *ADAMTS2* in SCZ. (4) Failure of some APDs to inhibit CREB activity and/or *ADAMTS2* mRNA expression in a D_1_ receptor-context. Our data herein do not discard that APDs might regulate *ADAMTS2* by indirect mechanisms like for example those involving β-arrestin, ERK or AKT signalling^42,48^.

In conclusion, we have confirmed the association between *ADAMTS2* expression and SCZ disease including its potential role in the clinical efficacy of APDs. Transcription of *ADAMTS2* is primarily controlled by the activity of D_1_-class receptors through cAMP/CREB and MAPK signalling. The unbiased investigation of the molecular mechanisms triggered by APDs, may provide a landscape of novel targets potentially associated with improved therapeutic responses.

## Supplementary information

Supplementary information

Supplementary Table S1

Supplementary Table S2

Supplementary Figure S1

Supplementary Figure S2

Supplementary Figure S3

Supplementary Figure S4

Supplementary Figure S5

Supplementary Figure S6
