# Supplementary material for: Dopaminergic control of ADAMTS2 expression through cAMP/CREB and ERK: molecular effects of antipsychotics
Source: Transl Psychiatry. 2019 Nov 18;9:306. doi: 10.1038/s41398-019-0647-7 (PMC6861307; doi:10.1038/s41398-019-0647-7)
Supplement: Supplementary file 1 — Supplementary information [file 41398_2019_647_MOESM1_ESM.docx]

**SUPPLEMENTARY INFORMATION**

**SUPPLEMENTARY MATERIAL AND METHODS**

**Human samples and Study Setting**

All referrals to *Programa Asistencial Fases Iniciales Psicosis* (PAFIP) were screened for patients who met the following criteria: 1) 15–60 years; 2) living in the catchment area (Cantabria); 3) experiencing their first episode of psychosis; 4) no prior regular treatment with antipsychotic medication or, if previously treated, a total lifetime of adequate antipsychotic treatment of less than 6 weeks; and 5) DSM-IV criteria for schizophrenia, schizophreniform disorder, schizoaffective disorder, or brief psychotic disorder.

Patients were excluded for any of the following reasons: 1) meeting DSM-IV criteria for drug dependence; 2) meeting DSM-IV criteria for mental retardation; or 3) having a history of neurological disease or head injury. The diagnoses were confirmed using the Structured Clinical Interview for DSM-IV, carried out by an experienced psychiatrist 6-months after the baseline visit.

Our operational definition for a first episode of psychosis included individuals with a non-affective psychosis (meeting the inclusion criteria defined above) who have not previously received antipsychotic treatment regardless of the duration of psychosis.

However, a subset of those individuals included in PAFIP was eligible for the present investigation. Thus, uniquely patients with a first episode of non-affective psychosis who had not previously taken antipsychotics (not even a single dose) at the time of baseline blood test and had mRNA samples at baseline and at 3-months were selected.

**Premorbid, sociodemographic variables and blood sample collection**

Premorbid and sociodemographic information was collected from patients, relatives and previous medical records (Table S2). In order to minimize the effects of diet and technique, blood samples were obtained from fasting subjects from 8:00 to 10:00 a.m. by the same medical staff, in the same setting. None of the patients had a chronic inflammation or infection or were taking medication that could directly influence the results of blood tests.

**RNA *in situ* hybridization images**

To reveal the localization of specific mRNA sequence of *ADAMTS2* expression on mouse embryo brain at stage E14.5, we take advantage of a RNA *in situ* hybridization (ISH) images collected in the Transcriptome Atlas Database for Mouse Embryo from Eurexpress project web page (Link: <http://www.eurexpress.org>), described elsewhere ^1^.

**Mice samples and immunohistochemical staining**

Five embryos at stage E18.5 and five postnatal P7 mice (C57BL/6) were anesthetized, perfused transcardially with PBS, followed by 4% PFA in PBS. Whole brain was analyzed in each case: brains were cut and mounted in 5 series, one of them was used to perform immunohistochemistry against ADAMTS2 (Santa Cruz biotechnology, CA, USA). Each series consisted of 8 slices for E18.5 and 12 for stage P7. Finally, between 48 (E18.5) and 72 (P7) brain sections were analyzed per animal. All animal experimental assays were performed in compliance with Spanish and European Union laws on animal care in experimentation (Council Directive 86/609/EEC) and approved by the Animal Experimentation Committee of our University.

Brains were removed and fixed overnight at 4°C (4% PFA). After dehydrated and paraffin-embedded, 10 µm coronal sections were mounted, de-waxed and rehydrated for immunostaining. Samples were rinsed with phosphate buffer solution (0.075 % Triton X-100, PBS-T), followed by a 1h incubation using a blocking solution (PBS-T and 10 % Goat serum). Sections were then incubated with the ADAMTS-2 primary antibody (Santa Cruz biotechnology, CA, USA), overnight at 4ºC and after were rinsed three times in PBS-T and incubated 1h with the appropriate secondary antibody. Finally, sections were washed and mounted on glass slides with 10:1 Mowiol (Calbiochem)-NPG (Sigma). Colorimetric detection was performed with 1% 3,39-Diaminobenzidine (DAB; Vector Laboratories SK-4100) and 0.0018 % H_2_O_2_ in PBS.

**SUPPLEMENTARY REFERENCES**

1. Diez-Roux G, Banfi S, Sultan M, Geffers L, Anand S, Rozado D*, et al*. A high-resolution anatomical atlas of the transcriptome in the mouse embryo. *PLoS Biol* 2011; **9**(1)**:** e1000582.

**SUPPLEMENTARY FIGURES AND TABLES LEGENDS**

**Table S1. Primer sequence of genes used in this study.** Gene ID: NCBI identification number; Ref Seq: NCBI Reference Sequence; F: forward primer sequence; R: reverse primer sequence; bp: base pair.

**Table S2. Demographic and clinical characteristics of the cohort of drug-naïve patients with a first-episode of psychosis.**

**Figure S1. Transcriptional control of ADAMTS2 using antipsychotic drugs.** Relative ADAMTS2 mRNA expression level in SK-N-SH cells incubated with risperidone (10 µM) for the indicated times (N=5). One-way ANOVA for multiple comparations. Data are mean ± S.E.M: *p < 0.05, **p < 0.01 vs control condition (vehicle, grey bars).

**Figure S2. Analysis of ADAMTS2 gene and protein expression profile in the mouse brain during development.** (A-D) Shows ADAMTS2 mRNA expression at stage E14.5 from Eurexpress data base (see methods). ADAMTS2 expression in the germinative neuroepithelium (Dgn) and migratory neural stream (Dms) of the dentate gyrus (arrows in A and C). In the progenitor cells (rhombic leap and migrator stream) of the external granular layer in the cerebellum (Cb). In the Ventral Tegmental Area (Atv), in the tegmental mesencephalon (Mes) (arrow in B and D), and caudally, in the progenitors of the isthmic organizer region (Iso) (arrowhead in D). (E-O) At E18.5 (E-I) and P7 ADAMTS2 protein was detected in the neuropiles of the subplate of anterior cingulate (CCx) and insular (ICx) cortex, lateral septum (Ls), endopiriform nucleus (EP) and striatal shell region (St) (E, F). In the hippocampus, ADAMTS2 was localized in the stratum lacunosum and moleculare of CA1 (sl-m) and proximal fimbria (fi) (G-I). At P7 ADAMTS2 protein (J-O) was detected in some cells and neuropile of the infrapyramidal blade of dentate gyrus (DGip) and its related molecular layer (ml) (J-L). Immunoreactivity was also detected in the neuropile of the lateral anterior hypothalamus (LA), with a strong expression in its limit with the suprachiasmatic nucleus (SCh) (M-O) (N=5, 48 for E18.5 and 72 for P7 sections per brain).

**Figure S3. Effects of selective antagonist over clozapine-mediated repression of ADAMTS2 mRNA expression.** Relative ADAMTS2 mRNA expression level in SK-N-SH cells pre-incubated 30 min with the indicated selective antagonist: SCH 39165 (D_1_-class receptors) (N=3), L 741,626 (D_2_-class receptors) (N=3), WAY 100635 (5-HT_1A_ receptor) (N=5) and MDL 100907 (5-HT_2A_ receptor) (N=6), and then incubated for 1 h with clozapine (N=4) (Drug concentration 1 µM). Student’s t-test: Data are mean ± S.E.M: *p < 0.05, **p < 0.01, ***p < 0.001 vs control condition (vehicle, grey bars), and #p < 0.05 vs clozapine condition (striped bar).

**Figure S4. Molecular mechanisms triggered downstream of D_1_ activation.** (A) ADAMTS2 mRNA levels by qRT-PCR in cells incubated for 1h with SKF 83822 (D_1_-class selective agonist; 1 µM) (N=4) and cholera toxin (CTX; Gαs subunit activator, 100 ng/ml) (N=3), or pre-incubated 30 min with CTX and then incubated with SKF 83822 for 1h (red bar) (N=4). (B) ADAMTS2 mRNA levels by qRT-PCR in cells incubated with SKF 83822 and pertussis toxin (PTX; Gαi subunit inhibitor, 50 ng/ml), or pre-incubated 30 min with PTX and then incubated with SKF 83822 for 1h (blue bar) (N=5). (C) ADAMTS2 mRNA levels by qRT-PCR in cells incubated for 1h with SKF 83822 and YM-254890 (Gαq subunit inhibitor, 1 µM), or pre-incubated 30 min with YM-254890 and then incubated with SKF 83822 for 1h (striped bar) (N=3). (D-F) Western-blots showing relative phosphorylation levels of CREB, ERK1/2 and p38 in SK-N-SH cells incubated with SKF 83822 (1 µM), as indicated for 15-, 30- and 60-min; graph bars indicate quantification of fold activation (N=3-5); one-way ANOVA for multiple comparations. And (G) Western-blots showing relative phosphorylation levels of PKA substrates in SK-N-SH cells incubated with SKF 83822 at the indicated times and agonist concentrations (N=1); pictures show a representative blot of each condition. Data are mean ± S.E.M: *p < 0.05, **p < 0.01, ***p < 0.001 vs control condition; and #p < 0.05 vs activation with SKF 83822.

**Figure S5. Transcriptional mechanisms that control ADAMTS2 gene expression downstream of dopamine D_1_-class receptors.** (A) *CREB1* knock-down SK-N-SH cells by transfection of stably expression shRNA and GFP reporter construct (orange bars), *CREB1* mRNA level (up) and CREB protein total expression (down) in scrambled (SCR) or shCREB1 cells (N=3). Stably *CREB1* Knock-down SK-N-SH cells were incubated for 1 h with SKF 83822 (1 µM, blue bars), forskolin (10 µM, red bars) and TPA (10 ng/ml, green bars): qRT-PCR showing *ADAMTS2* (B) and *C-FOS (C)* mRNA expression in shCREB1 or non-targeted control (NTC) cells (N=3-4). And (D) stably expressing shCREB1 or scrambled (SCR) SK-N-SH cells incubated for 15 min with SKF 83822 (1 µM) (N=3). All cells were selected with puromycin (1 µg/ml) at least 7 days. Images are representative of independent experiments. Data are mean ± S.E.M; Student’s t-test: **p < 0.01, ***p < 0.001 shows significance with respect to control (Vehicle); and #p < 0.05, ###p < 0.001 vs each condition in SCR cells.

**Figure S6. PKA-dependent and independent signalling activation.** (A) Western-blots showing relative phosphorylation levels of PKA substrates in SK-N-SH cells treated with forskolin (10 µM) and TPA (10 ng/ml), at the indicated time points (N=1). And relative phosphorylation levels of CREB (B) and ERK1/2 (C) in SK-N-SH cells incubated with forskolin (10 µM), at the indicated times (N=3). Images are representative of each western-blot. Data are mean ± S.E.M; Student’s t-test: *p < 0.05, ***p < 0.001 vs 0 h.
