# Supplementary figures and images for: Dopaminergic control of ADAMTS2 expression through cAMP/CREB and ERK: molecular effects of antipsychotics
Source: Transl Psychiatry. 2019 Nov 18;9:306. doi: 10.1038/s41398-019-0647-7 (PMC6861307; doi:10.1038/s41398-019-0647-7)

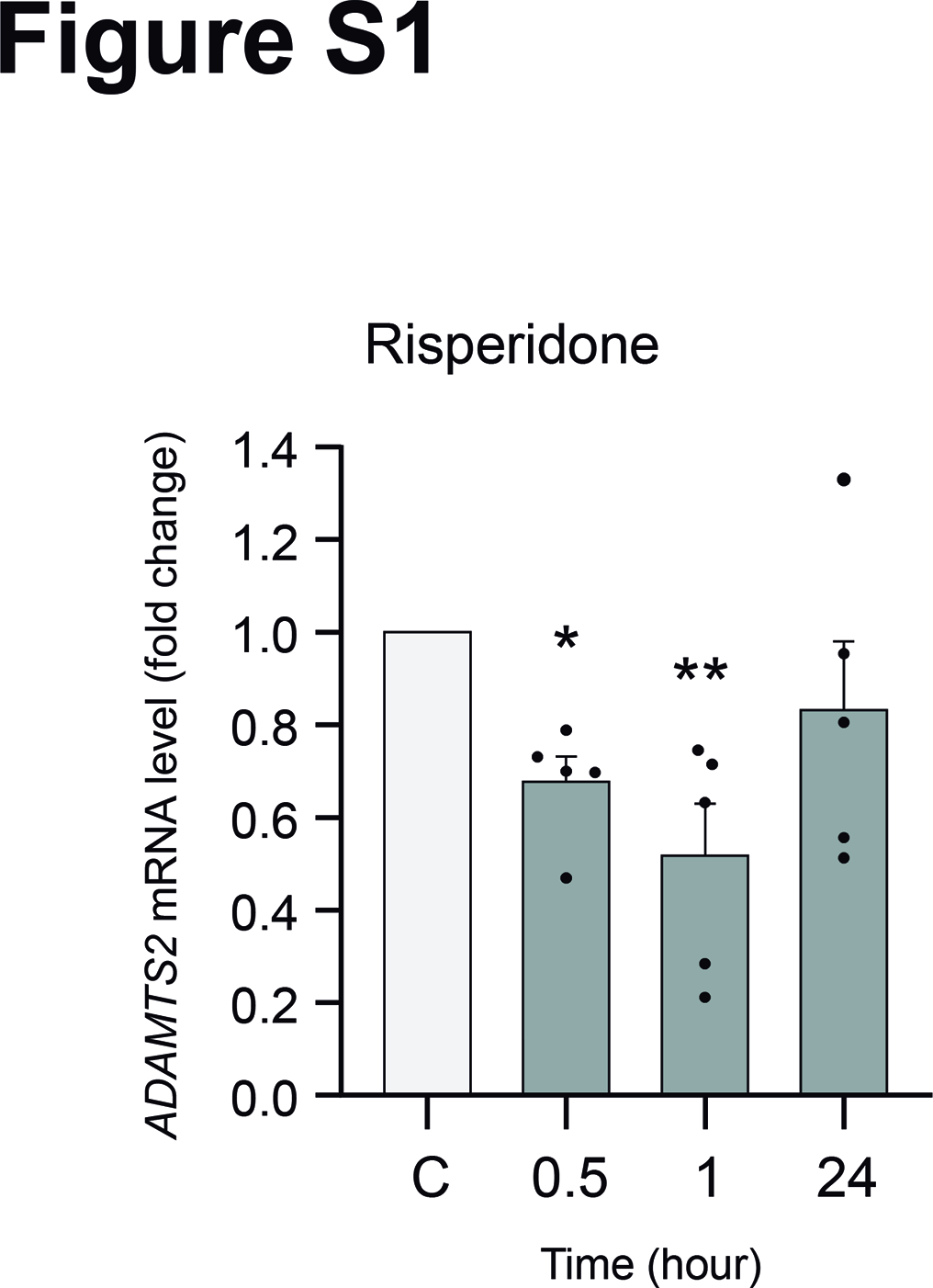

Supplement: Supplementary file 4 — Supplementary Figure S1 [file 41398_2019_647_MOESM4_ESM.tif]

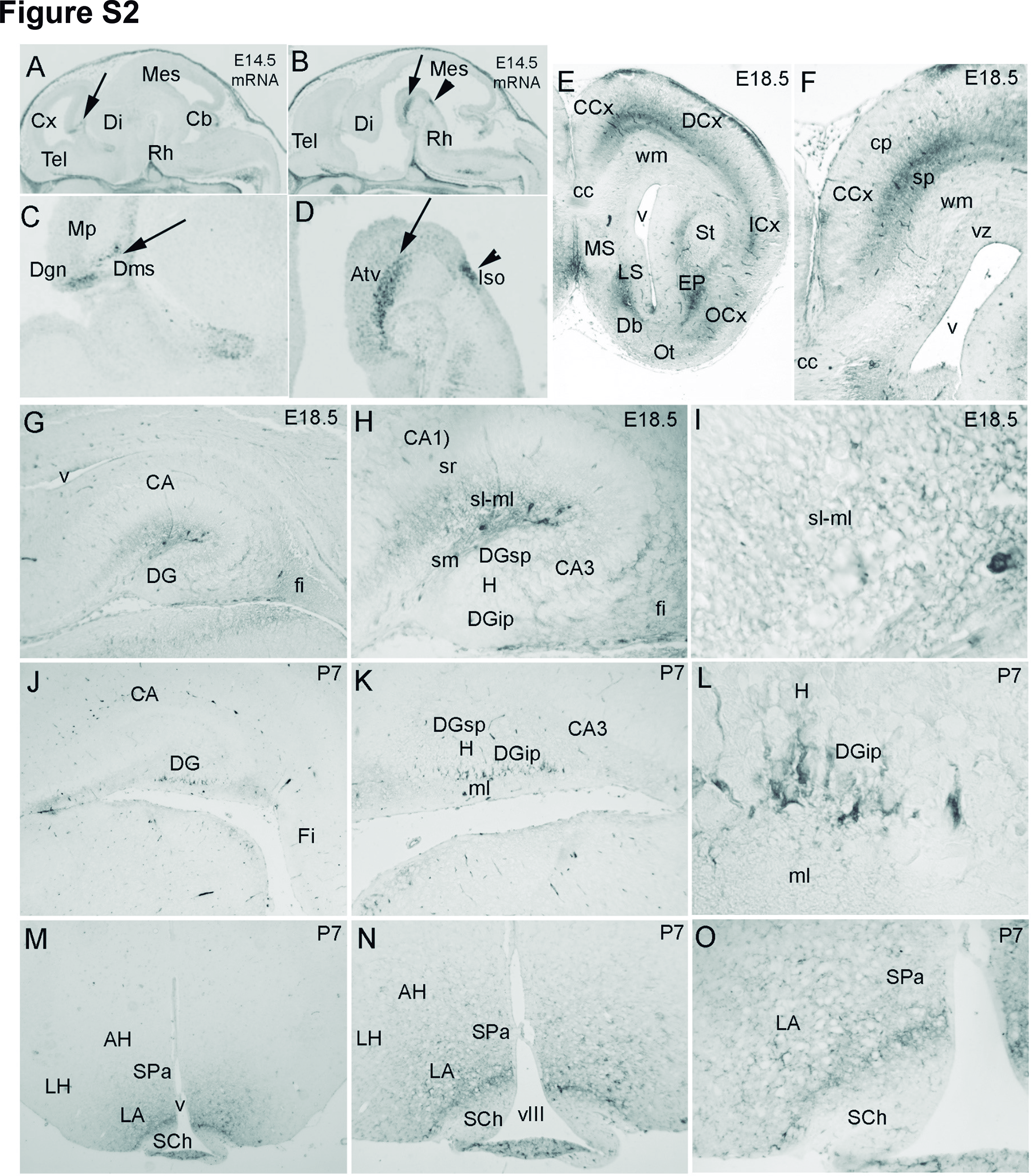

Supplement: Supplementary file 5 — Supplementary Figure S2 [file 41398_2019_647_MOESM5_ESM.tif]

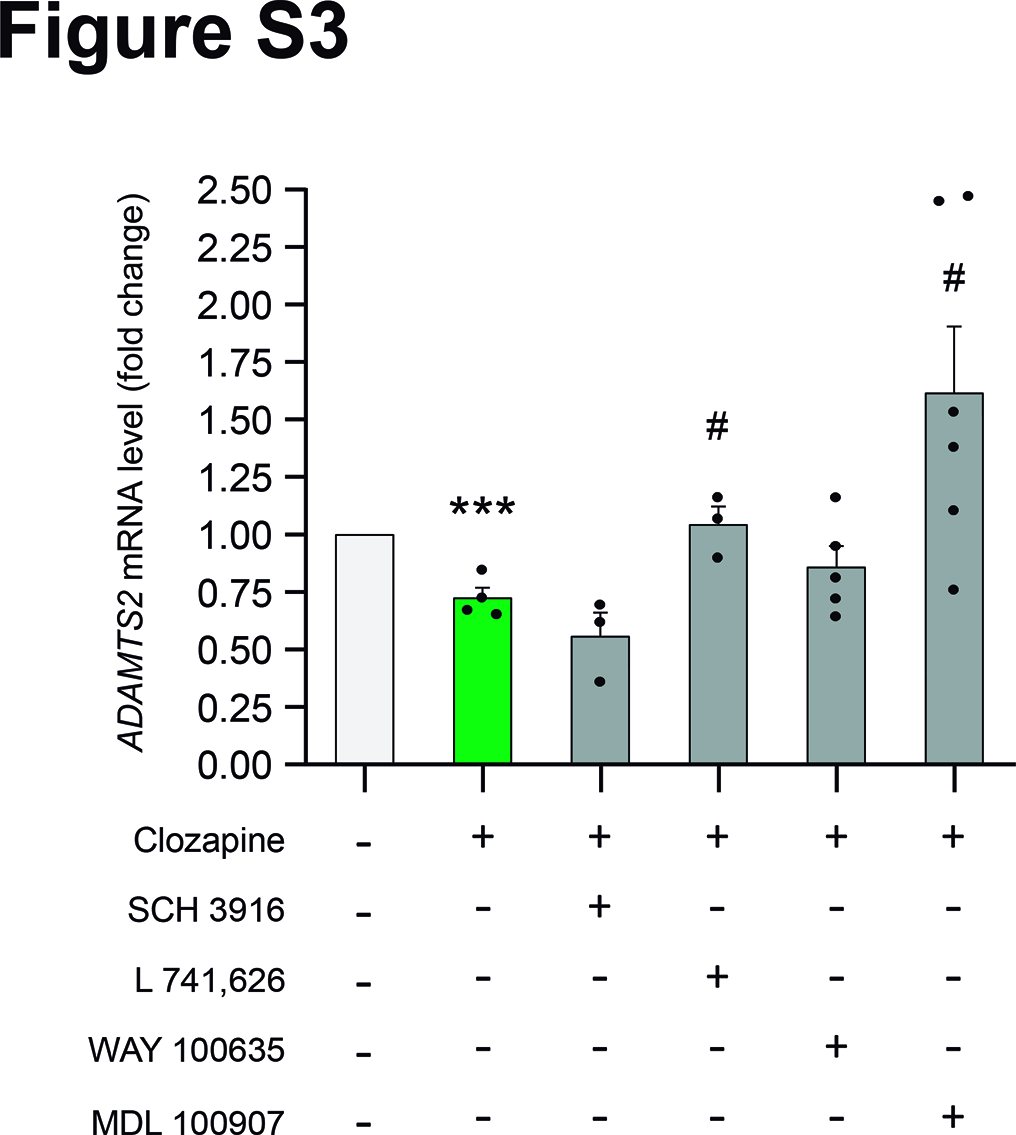

Supplement: Supplementary file 6 — Supplementary Figure S3 [file 41398_2019_647_MOESM6_ESM.tif]

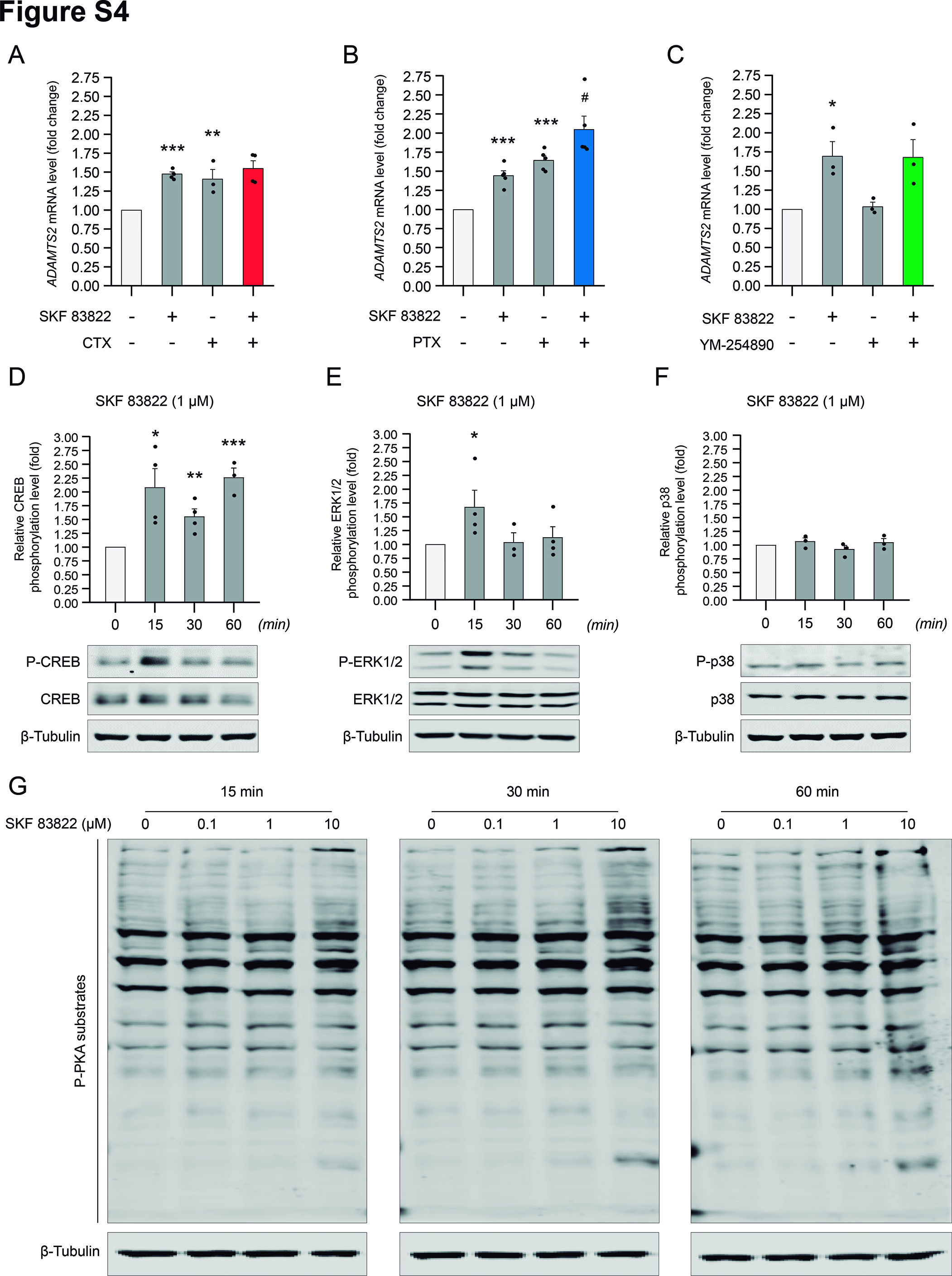

Supplement: Supplementary file 7 — Supplementary Figure S4 [file 41398_2019_647_MOESM7_ESM.tif]

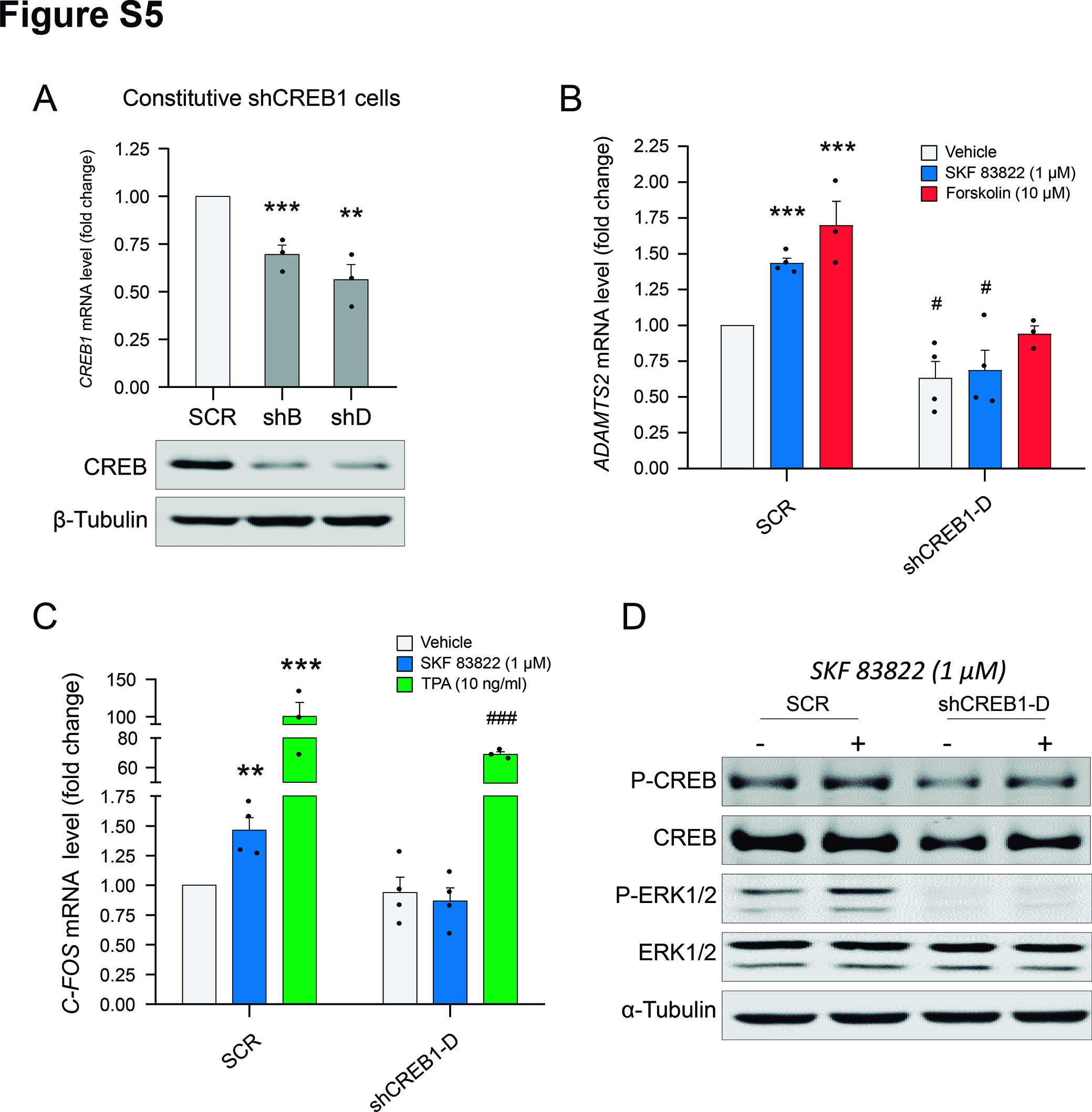

Supplement: Supplementary file 8 — Supplementary Figure S5 [file 41398_2019_647_MOESM8_ESM.tif]

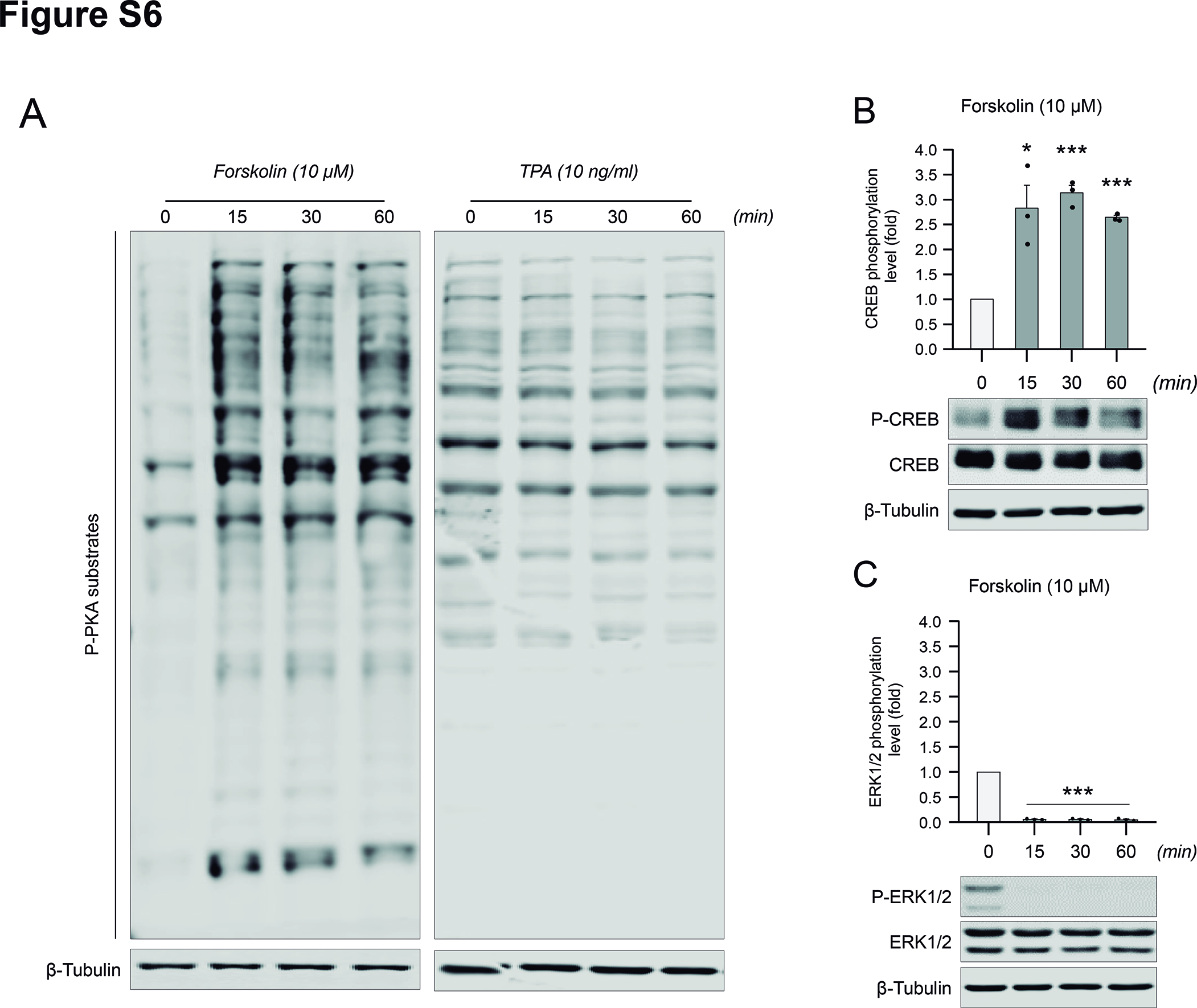

Supplement: Supplementary file 9 — Supplementary Figure S6 [file 41398_2019_647_MOESM9_ESM.tif]
